# The Responses of *Lactobacillus reuteri* LR1 or Antibiotic on Intestinal Barrier Function and Microbiota in the Cecum of Pigs

**DOI:** 10.3389/fmicb.2022.877297

**Published:** 2022-06-02

**Authors:** Bijing Yang, Chunyan Liu, Yanna Huang, Qiwen Wu, Yunxia Xiong, Xuefen Yang, Shenglan Hu, Zongyong Jiang, Li Wang, Hongbo Yi

**Affiliations:** ^1^State Key Laboratory of Livestock and Poultry Breeding, Ministry of Agriculture Key Laboratory of Animal Nutrition and Feed Science in South China, Guangdong Key Laboratory of Animal Breeding and Nutrition, Guangdong Laboratory for Lingnan Modern Agriculture, Institute of Animal Science, Guangdong Academy of Agricultural Sciences, Guangzhou, China; ^2^College of Veterinary Medicine, South China Agricultural University, Guangzhou, China; ^3^College of Animal Science and Technology, Guangxi University, Nanning, China

**Keywords:** cecum, microbiome, intestinal barrier function, antibiotics, *Lactobacillus reuteri*, pig

## Abstract

This study aimed to investigate responses of the *Lactobacillus reuteri* or an antibiotic on cecal microbiota and intestinal barrier function in different stages of pigs. A total of 144 weaned pigs (*Duroc* × *Landrace* × *Yorkshire*, 21 days of age) were randomly assigned to the control group (CON, fed with a basal diet), the antibiotic group (AO, fed with basal diet plus 100 mg/kg olaquindox and 75 mg/kg aureomycin), and the *L. reuteri* group (LR, fed with the basal diet + 5 × 10^10^ CFU/kg *L. reuteri* LR1) throughout the 164-d experiment. A total of 45 cecal content samples (5 samples per group) from different periods (14th, 42th, and 164th days) were collected for 16S rRNA gene amplification. The results revealed that although LR and AO did not change the diversity of cecal microbiota in pigs, the abundance of some bacteria at the genus level was changed with age. The proportion of *Lactobacillus* was increased by LR in early life, whereas it was decreased by AO compared with the control group. The relative abundance of *Ruminococcaceae* was increased along with age. In addition, the gas chromatography results showed that age, not AO or LR, has significant effects on the concentrations of SCFAs in the cecum of pigs (*P* < 0.05). However, the mRNA expression of tight junction proteins *zonula occluden-1* (*ZO-1*) and *occludin* were increased by AO in the cecum of pigs on day 14, while LR increased the mRNA expression of intestinal barrier-related proteins *ZO-1*, *occludin*, *mucin-1*, *mucin-2*, *PG1-5*, and *pBD2* in the cecum of pigs on days 14 and 164 (*P* < 0.05). In conclusion, LR and AO have different effects on the intestinal barrier function of the cecum, and neither LR nor AO damaged the intestinal barrier function of pig cecum. In addition, LR and AO have little effects on cecal microflora in different stages of the pigs. The microflora and their metabolite SCFAs were significantly changed along with age. These findings provide important information to understand the homeostasis of the cecum of pigs after antibiotic or probiotic treatment.

## Introduction

Antibiotics are a means of maintaining and improving the health of animals. However, the complexity and diversity of intestinal microbiome and differences in intestinal environment and antibiotic treatment have a great difference in the impact of intestinal microbiome and immune response (Mu et al., 2017). On the one hand, antibiotics can accelerate the development and maturation of the gut microbiome (Kim et al., 2012), and improve feed conversion to promote the growth of livestock and poultry (Looft et al., 2012). In addition, antibiotics are also used for treatment and prevention of diseases. It has been reported that antibiotics can reduce necrotizing enterocolitis in premature piglets by regulating intestinal immunity (Jensen et al., 2014). On the other hand, as antibiotics are overused, the risk of spreading antimicrobial resistance has also increased (Mencia-Ares et al., 2020). Herrero-Fresno et al. (2016) found that the prevention of diarrhea in piglets by ampramycin may lead to cross-resistance of *Escherichia coli* and *Streptococcus* to apramycin/gentamicin.

As antibiotic resistance has become a serious public health problem, probiotics have been found as an alternative to antibiotics (Shin et al., 2019). *Lactobacillus reuteri* is one of the main species of probiotics in the gastrointestinal tract of pigs. Previous studies have demonstrated that porcine *L. reuteri* improved growth performance and intestinal barrier function by regulating the composition of intestinal microbial community in pigs (Liu et al., 2017; Yi et al., 2018).

The gut microbiota of mammalian is very complex and plays an important role in maintaining intestinal morphology, nutrient digestion, and immune regulation (Luo et al., 2015). Previous studies have shown differences in the distribution of microbiota in the small and large intestines of pigs (Mu et al., 2017). In addition, the stable microbial community in the intestine is the precursor for the host to exert normal physiological functions (Dabke et al., 2019). The cecum, as the transition between the small intestine and the large intestine, plays an important role. For example, some nutrients cannot be fully digested in the small intestine and need to be fermented in the cecum to produce short-chain fatty acids (Tan et al., 2017). There are few studies on cecal microbiota of pigs, presently. Furthermore, the effects of antibiotics and *L. reuteri* on the cecal microbiota and intestinal barrier function are still unclear. Therefore, we explored the effects of antibiotics and *L. reuteri* on the development and composition of the cecal microbiota and intestinal barrier function in different stages of pigs.

## Materials and Methods

All animal experimental protocols used in this study were according to the Chinese guidelines for animal welfare and approved by the Animal Care and Use Committee of Guangdong Academy of Agricultural Sciences.

### Animal Treatment and Sample Collection

A total of 144 weaned pigs (*Duroc* × *Landrace* × *Yorkshire*, an initial BW of 6.49 ± 0.02 kg, 21 days of age) were balanced for sex and then randomly assigned to 3 dietary treatments with 8 replicate pens per treatment, with each pen containing 6 piglets. The three experimental treatments were briefly described as follows: the control (CON) group, which was fed with a basal diet; the antibiotic group (AO) group, which was fed with the basal diet + 100 mg/kg olaquindox + 75 mg/kg aureomycin, and *L. reuteri* (LR) group, which was fed with the basal diet + 5 × 10^10^ CFU/kg *L. reuteri* LR1. The basal diet was formulated according to the nutritional requirements of pig body weight (NRC, 2012), and the components and composition of the basal diet are shown in Supplementary Table 1. The experimental site was located at the Institute of Animal Science, Guangdong Academy of Agricultural Sciences. The house was completely enclosed with temperature control devices. The pigs were raised in leaky pens off the ground and fed 4 times a day at 08:00 am, 12:00 am, 4:00 pm, and 8:00 pm. The pigs were free to feed and drink water during the experiment.

The 14th and 42nd days after weaning are important nodes in the growth stage of pigs, and the 164th day is the day of slaughter. Therefore, on the 14th(S1), 42th(S2), and 164th (S3) days after weaning, one pig was selected randomly from each pen and then anesthetized and sacrificed. Samples of cecal content were collected and stored at –80°C. A section of the cecum sample was washed with PBS and fixed in paraformaldehyde (4%). Another part of the cecum sample was rinsed thoroughly with normal saline, collected, and stored at –80°C.

### Histopathological Examination

Referring to our previous method (Feng et al., 2021), in short, a cecal tissue was fixed with 4% paraformaldehyde, followed by gradient dehydration and embedding, and then stained with hematoxylin and eosin. We measured the length of mucous and muscularis, submucosa, and muscularis of the cecum and analyzed them.

### Real-Time Polymerase Chain Reaction Analysis

RNA was extracted from the cecum of the pig using a TRIzol reagent (Invitrogen, Carlsbad, CA) and the extracted RNA was reverse-transcribed into cDNA using PrimeScript RT Reagent Kit (Takara, Dalian, China). Then, real-time PCR was performed using a CFX Connect Detection System (Bio-Rad, Hercules, CA, United States), and the thermocycler protocol refer to our previous methods (Yi et al., 2018). The primers used for real-time polymerase chain reaction (PCR) are summarized in Supplementary Table 2, and *GAPDH* was used as the reference gene. Quantitative detections of total bacteria (forward: CGGTGAATACGTTCYCGG; reverse: GGWTACCTTGTTACGACTT), *Escherichia coli* (forward: CATGCCGCGTGTATGAAGAA; reverse: CGGGTAACGTCAATGAGCAAA), *Lactobacillus* (forward: CGATGAGTGCTAGGTGTTGGA; reverse: CAAGATGTCA AGACCTGGTAAG) were performed by qPCR using the StepOne PlusTM System.

### Immunofluorescence

For immunofluorescence analysis, paraffin-embedded sections of the cecum were incubated at 37°C for 24h. Then, the sections were dewaxed, rehydrated, and treated with 1% Triton X-100 for 30 min. The sections were sealed with serum and then incubated overnight with primary antibody (anti-occludin, GeneTex Inc., Irvine, CA) at 4°C. After incubation with a secondary antibody (Abcam), the sections were stained with DAPI (China, Beyotime). Images were obtained using an ECLIPSE TI-SR microscope with a DS-U3 Image-Pro system (Nikon, Tokyo, Japan).

### 16s rRNA Gene Amplification and Illumina MiSeq Sequencing

Total genomic DNA from the samples of cecal content was extracted using the CTAB/SDS method, and the concentration and quality were evaluated with Qubit 2.0 Fluorometer (Invitrogen, Carlsbad, CA) and agarose gel. We used 30–50 ng DNA as a template, which included the “CCTACGGRRBGCASCAGKVRVGAAT” sequence upstream primer and the “GGACTACNVGGGTWTCTAATCC” sequence downstream primer amplification V3 and V4 areas. In addition, a connector with an index was added to the end of PCR products of 16S rDNA by PCR for NGS sequencing. Quality inspection of sequencing libraries was conducted using an Agilent 2100 bioanalyzer (Agilent Technologies, Palo Alto, CA, United States), and sequencing library concentrations were measured with Qubit2.0 Fluorometer (Invitrogen, Carlsbad, CA). After mixing the DNA library, the 2 × 300 bp dual-end sequencing (PE) was performed according to the Illumina MiSeq (Illumina, San Diego, CA, United States) instrument instructions, and the sequence information was read with the MiSeq Control Software (MCS) supplied with Miseq.

### Bioinformatics Analysis

The forward and reverse reads obtained by paired-end sequencing are first assembled and connected in pairs; sequences containing N in the splicing result are filtered, and sequences with a sequence length greater than 200 bp are retained. After quality filtering, the chimera sequence was removed, and the final sequence was used for OTU analysis. VSEARCH (1.9.6) was used for sequence clustering (sequence similarity was set to 97%). The 16S rRNA reference database for comparison was Silva 123. Then, the representative sequences of OTUs were analyzed by species taxonomy using the RDP classifier (Ribosomal Database Program) algorithm, and the community composition of each sample was counted under different species classification levels. Based on the analysis results of OTUs, the method of random sampling of the sample sequence was adopted to calculate alpha diversity indexes, such as Shannon and Chao1, and the dilution curve was drawn. The distance matrix between samples based on Brary-Curtis was used for PCoA (principal coordinates analysis) visualization to show beta diversity. Qiime (1.9.1) was used for the taxonomic analysis of OTU representative sequences at 97% similar levels, and the community composition of each sample was counted at each level. Differentially represented bacterial taxa between different samples were analyzed using the linear discriminant analysis effect size (LEfSe). The original sequence data were submitted to the Sequence Read Archive (SRA; NCBI, United States) with accession no. PRJNA768304.

### Determination of Short-Chain Fatty Acids in Samples

Referring to our previous method (Yi et al., 2016), 1 g cecal content was mixed with 1 ml double steam water and centrifuged at low temperature and high speed for 15 min to get the supernatant. Then, the supernatant was mixed with 85% orthophosphoric acid (20 μl/ml) and placed at 4°C for 1 h. Finally, the supernatant was obtained by centrifugation and transferred to gas chromatographic vials to detect the concentration of SCFAs with GC-8A (Shimadzu, Kyoto, Japan).

### Statistical Analyses

For statistical analysis, the SPSS 19.0 software (SPSS Inc., Chicago, IL) was used in this experimental research. All the collected data were analyzed by one-way analysis of variance (ANOVA) with Tukey’s test. All the data were expressed as means ± SEM. The differences were significant at *P* < 0.05.

## Results

### Effects of AO and LR on the Morphology of Cecum

**FIGURE 1:**
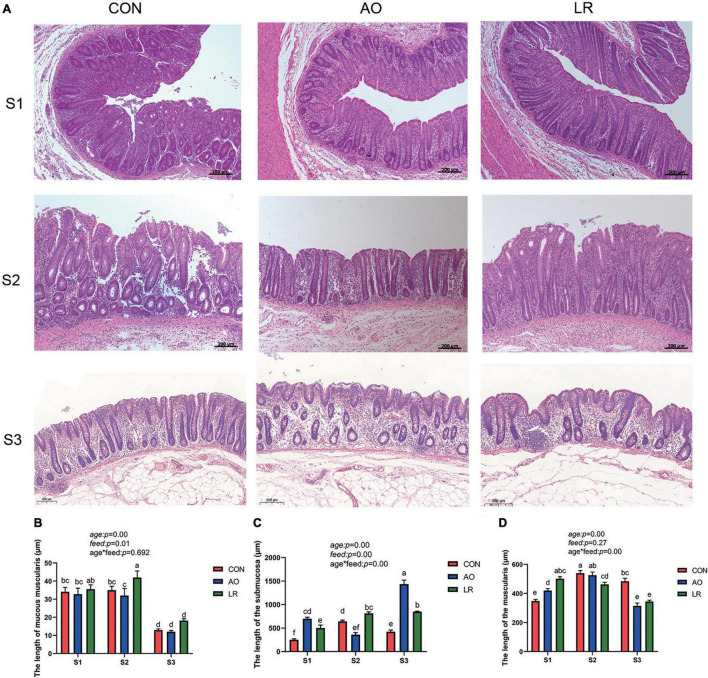
**(A)** Morphology of the cecum was observed by HE staining in different stages. **(B–D)** Length of muscularis, submucosa, and muscularis of the cecum. The *P* values indicate main effects for age, diet feed level, and their interaction. All data are expressed as the mean ± SEM. ^a,b,c,d,e,f^Means lacking common superscript letter indicated significant differences (*P* < 0.05).

We observed the morphology of the cecum by hematoxylineosin stain (Figure 1A), and the length of the mucosae muscularis, submucosa, and muscularis of the cecum was measured (Figures 1B–D). The results showed that age had a significant effect on muscularity, submucosa and muscularity (age: *p* < 0.05). The diet feed with AO and LR had significant effects on mucosal musculature and submucosa (feed: *p* < 0.05), which had no significant effect on muscle layer (feed: *p* = 0.27). In addition, there was an interaction between age and diet on the submucosal and muscular layers (age*feed: *p* < 0.05).

### Effects of AO and LR on Cecal Barrier Function

**FIGURE 2:**
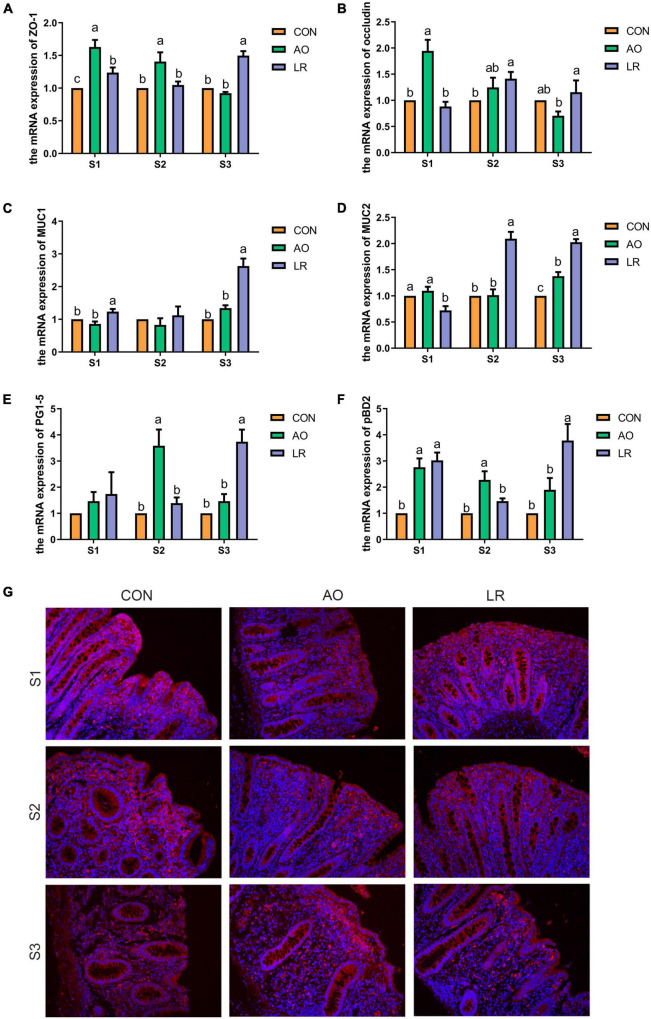
Expression of barrier function-related genes in the cecum. **(A–F)** Relative mRNA expression levels of *ZO-1*, *occludin*, *MUC1*, *MUC2*, *PG1-5*, and *pBD2*. **(G)** Immunofluorescence images of cecal paraffin section occludin. All data are expressed as the mean ± SEM. ^a,b,c^Means lacking common superscript letter indicated significant differences (*P* < 0.05).

To assess the effects of AO and LR on cecal barrier function, the expression of the tight junction (TJ) gene was investigated by q-PCR (Figures 2A,B). The results revealed that the level of expression of the mRNA of *occludin* and *zonula occludens* (*ZO-1*) was significantly increased in the AO group at 14 days compared with the other groups *(p* < *0.05)*. At 164 days, the expression of *ZO-1* and *occludin* was significantly increased in the LR group compared with the AO group *(p* < 0.05*)*. The results are shown in Figures 2C,D; compared with the other groups, the level of expression of mRNA of *mucin1* (*MUC1*) and *mucin2* (*MUC2*) was significantly increased in the LR group on day 164 *(p* < 0.05*)*. In addition, we verified the expression of *occludin* in the cecum by immunofluorescence (Figure 2G). With regard to antimicrobial peptides (Figures 2E,F), the results showed that on day 42, compared with the other groups, the level of expression of mRNA of *protegrin* (*PG1-5*) and porcine antibacterial peptide β*-Defensin-2* (*pBD2*) in the AO group was significantly increased *(p* < 0.05*)*, and on day 164, the level of expression of *PG1-5* and *pBD2* in the LR group was significantly increased compared with the AO group *(p* < 0.05*).*

### Sequences Analyses

**FIGURE 3:**
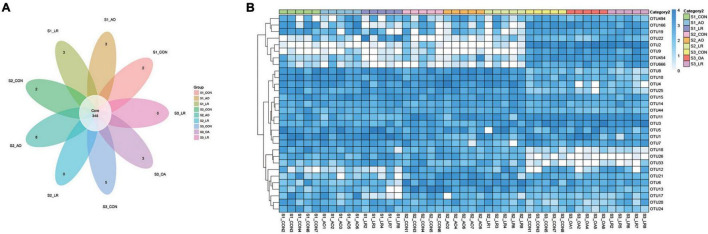
Operational taxonomic units (OTUs). **(A)** Venn diagrams for core OTU distribution in the cecum. **(B)** Heatmap of the 30 most abundant OTUs in the cecum.

In the microbiome analysis, a total of 1,300,105, 1,282, 244, and 1,271,917 original sequences were acquired from S1, S2, and S3, respectively (Supplementary Table 3). After optimizing the original data, a total number of 3,204,268 effective sequences were obtained from all the samples. Moreover, we performed an OUT division of all sequences based on 97% similarity of nucleotide sequences and observed 348 core OTUs in the cecum (Figure 3A). To further demonstrate the similarity and difference of the samples, we analyzed the 30 OTUs with highest abundance with a heat map (Figure 3B). In addition, the rank-abundance curve of each sample is wide and smooth, indicating good species evenness and richness (Supplementary Figure 1B). We randomly sampled the sequences and constructed a rarefaction curve based on the number of extracted sequences and the number of OTUs they could represent. The number of qualified sequences per sample reached more than 10,000, showing a saturation trend and indicating the rationality of sequencing data volume (Supplementary Figure 1A).

### Alterations in Cecal Microbial Diversities With the Effect of AO and LR

**FIGURE 4:**
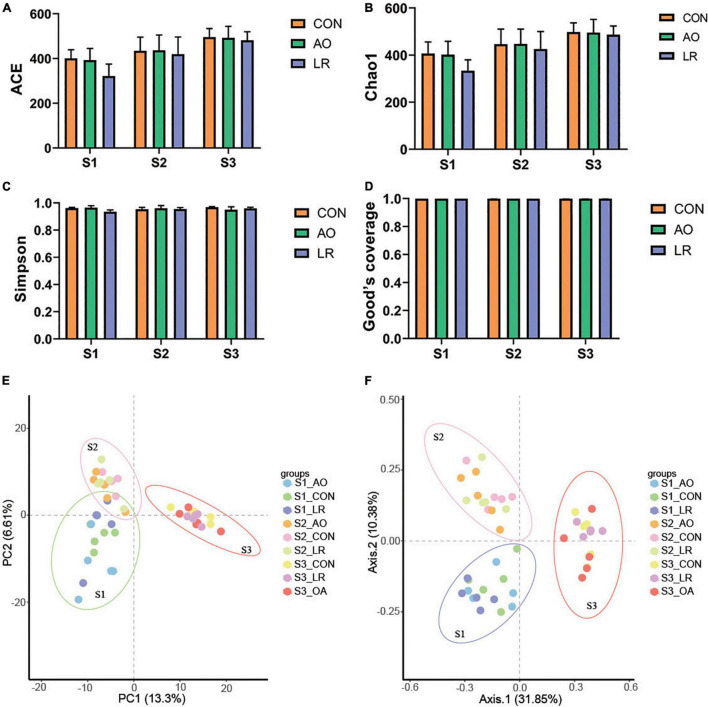
Microbial diversity index analysis. Gut bacterial alpha diversity can be determined by **(A)** ACE, **(B)** Chao1, **(C)** Simpson, and **(D)** Good’s coverage. **(E,F)** Principal component analysis (PCA) and principal coordinates analysis (PCoA) of cecal microbiota in the three groups. Each dot represents a sample, and same color indicates same group.

To evaluate the diversity and abundance differences in cecal microbiota between the groups, alpha and beta indices were estimated by comparing qualified sequences. The diversity of alpha in gut microbial community can be reflected by the indexes of ACE, Chao1, Simpson, and Good’s Coverage. The results showed that sample sequencing depth was estimated to be about 100%, indicating high sample coverage (Figure 4D). Compared with the other groups, the index of ACE and Chao1 were highest in the control group, while the ACE and Chao1 indexes in the LR group were the lowest. We observed that with change in age, the ACE and Chao1 indexes in the all groups showed an upward trend, but that there was no significant difference (Figures 4A,B). Moreover, there was no distinct difference in Simpson index (Figure 4C). The results revealed that AO and LR administration had no effect on the abundance and diversity of cecal microflora of the pigs.

Principal component analysis and principal coordinates analysis (based on Brary-Curtis distance matrix method) were performed. We found that despite being fed with different diets, the pigs in the control group, the AO group, and the LR group were clustered together, indicating that the intestinal microbiota composition among the three groups in different stages was similar (Figures 4E,F). In addition, the PCA and PCoA results of different periods are shown in the Supplementary Figures 2A–F. Interestingly, the cecal microbiome of the pigs continued to change with age. These results revealed that AO and LR have no effect on the major composition of the cecum microbiota of pigs.

### The Cecal Microbiota Compositions in the Three Treatment Groups

**FIGURE 5:**
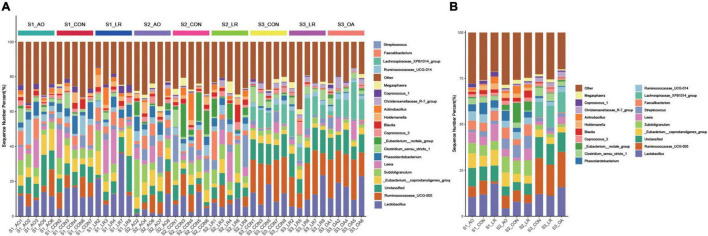
Relative abundance of intestinal microorganisms. **(A)** Relative abundance of the most dominant cecal microbiota in each sample at the genus (top 20) level. **(B)** Relative abundance of cecal microbiota on the basis of the average number of each subfamily at the genus levels.

**FIGURE 6:**
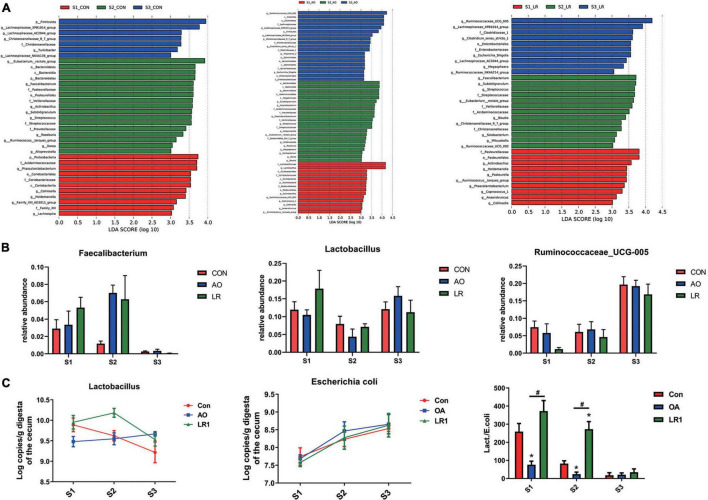
Analysis of differences between groups. **(A)** The LEfSe analysis (LDA score ≥ 3.5) identified the biomarker bacterial species in different groups. **(B)** Flora that differs at the genus level. **(C)** Quantitative polymerase chain reaction results of putrescine on cecal *Lactobacillus* and *Escherichia* coli and their ratio. *P < 0.05, ^#^*P* < 0.05.

We evaluated the relative proportion of dominant microbiota at the phylum and genus levels. The result of allocation by phylum is exhibited in Supplementary Figures 3A,B; *Firmicutes* was the most important bacteria in the cecum of pigs, accounting for more than 80% of the total sequence regardless of changes in feed and age. *Proteobacteria, Bacteroidetes, and Actinobacteria* were represented with a lower abundance in all the three groups, but they were also important floras in the cecum. At the genus level (Figures 5A,B), *Lactobacillus* was the most predominant bacteria in the cecum on day 14, except for other bacteria. On days 14 and 42, compared with the control group (11.92 and 7.96%), the abundance of *Lactobacillus* (10.46 and 4.35%) in the AO group was decreased. Interestingly, on day 164, compared with the control group (12.06%), the abundance of *Lactobacillus* was increased in the AO group (15.8%), while it was decreased in the LR group (11.21%). Moreover, compared with *Lactobacillus* (7.15 and 11.21%) abundance on days 42 and 164, the abundance of *Lactobacillus* (17.83%) in the LR group was highest on day 14. *Subdoligranulum* (8.68 and 8.33%) was the most dominant bacteria in the cecum of the 42-day AO and LR groups, except for other bacteria. The proportion of *Ruminococcaceae_UCG-005* was increased with age. However, the abundance of *Ruminococcaceae_UCG-005* in the LR group was lower than that in the control group regardless of age. Moreover, *Ruminococcaceae_UCG-005* (19.22 and 16.84%) was the most dominant bacterium in the cecum of the AO and LR groups at 164 days except for other bacteria. On day 164, the *Lachnospiraceae_XPB1014_*group appeared, and the AO and LR groups (11.04 and 11.64%) had an increase in the relative abundance of the *Lachnospiraceae_XPB1014_*group compared to the control group (8.37%). The LEfSe analysis identified discriminative species among the different groups (Figure 6A). We found differences in the flora in different stages, and the result is shown in Figure 6B. Moreover, we detected the expression of *Lactobacillus* and *Escherichia coli* by q-PCR (Figure 6C). We observed a significant decrease in the ratio of *Lactobacillus* to *E. coli* in the AO group on days 14 and 42 compared to the control group. The ratio of the LR group was significantly higher compared to the AO group.

### Effects of AO and LR Feeding on Cecal Fermented Metabolites of Pig

The main microbial products of intestinal fermentation are short-chain fatty acids (SCFAs). The results of AO and LR fermentation metabolites in the cecal digestive system are presented in Table 1. The concentrations of acetic acid were always the highest and isobutyric acid the lowest in cecum despite of different ages and diets. Compared with the control group, the volatile fatty acid (VFA) concentration of the AO group and the LR group was lower on days 14 and 42, but it was not significant. Interestingly, on day 164, compared with the control group, the total VFA concentration of the AO group and the LR group was increased but not significantly. These results demonstrated that dietary AO and LR have no significant effects on the concentrations of SCFAs in pigs, but that the age of pigs has significant effects on the concentrations of acetate, butyric acid, isobutyric acid, isovaleric acid, pentanoic acid, and total VFAs *(p* < 0.05).

**TABLE 1:** Quantitation of short-chain fatty acids in cecal contents.

	Items	Acetate	Propionic acid	Isobutyric acid	Butyric acid	Isovaleric acid	Pentanoic acid	Total VFA
	S1-CON	99.12^ab^	42.7	2.03^a^	18.55^ab^	2.35^ab^	2.98^bc^	167.73^ab^
	S1-AO	93.33^ab^	44.86	1.84^ab^	18.23^ab^	2.14abc	3.01^bc^	163.40^ab^
	S1-LR	90.15^ab^	46.07	1.89^a^	14.63^b^	2.44^a^	2.98^bc^	158.15^ab^
	S2-CON	111.70^a^	53.28	1.84^ab^	25.76^a^	2.52^a^	5.37^a^	200.46^a^
	S2-AO	97.89^ab^	47.54	2.09^a^	22.30^ab^	2.76^a^	4.73^ab^	177.31^ab^
	S2-LR	98.34^ab^	51.6	2.10^a^	20.60^ab^	2.73^a^	6.30^a^	181.66^ab^
	S3-CON	74.80^b^	40.87	1.00^c^	15.30^b^	1.19^c^	3.21^bc^	136.37^b^
	S3-AO	82.83^b^	40.79	1.07^c^	15.53^b^	1.28^c^	2.31^c^	143.81^b^
	S3-LR	80.40^b^	42.31	1.13^bc^	13.86^b^	1.44^bc^	2.87^bc^	142.02^b^
	SEM	3.15	1.79	0.09	1.09	0.12	0.39	5.51
*P*-valve	F	0.465	0.824	0.657	0.168	0.464	0.771	0.555
	D	0.007	0.092	0.001	0.006	0.001	0001	0.002
	F*D	<0.001	<0.001	<0.001	<0.001	<0.001	<0.001	<0.001

*^a,b,c^Means lacking common superscript letter indicated significant differences (P < 0.05) within a row.*

## Discussion

The intestinal health of pigs is one of the important factors affecting the development of the pig industry, and intestinal microbes play an essential role in it. Previous research have shown that porcine intestinal microflora show age-dependent maturation (Yu et al., 2018). Antibiotics and probiotics are also widely used in the pig industry. In this study, we investigated whether antibiotics and *L. reuteri* impair or improve cecal barrier function, and analyzed the effects of antibiotics and *L. reuteri* on the cecal microbiome, as well as the effects of age on them.

In this study, AO and LR changed the length of the mucosae muscularis, submucosa, and muscularis of the cecum. The intestinal mucosal barrier is maintained by tight junctions (TJs), and the main components of TJs are composed of *occludin*, *ZO-1*, and *claudin-1* (Forster, 2008). Additionally, intestinal epithelial surfaces are covered with mucin, which is one of the defensive measures against pathogen invasion (Wang et al., 2018). Our results revealed that the level of expression of the mRNA of *occludin* and *ZO-1* was significantly increased in the AO group on day 14 compared with the other groups. On day 164, the expression of *ZO-1*and *occludin* was significantly increased in the LR group compared with the AO group. The level of expression of the mRNA of *MUC1* and *MUC2* was significantly increased in the LR group on day 164. Wang and Yang et al. showed that *L. reuteri* significantly increased the expression of *occludin*, *ZO-1*, and *MUC2* in the intestinal epithelium of pigs (Yang et al., 2015; Wang et al., 2020), which is consistent with our results. Notably, previous studies have shown that antibiotic treatment leads to down-regulation of tight junction protein expression in the ileum of mice and damages the intestinal barrier (Feng et al., 2019), which is inconsistent with our results. This could be the result of species differences and location of the gut. In addition, we observed that on day 164, the level of expression of *PG1-5* and *pBD2* in the LR group was significantly increased. Liu et al. showed that *L. reuteri* can stimulate the expression of *pBD2* and *PG1-5* in the colon and enhance the expression of endogenous host defense peptides (HDP), thereby strengthening the mucosal antibacterial barrier of newborn piglets (Liu et al., 2017).

Mammalian gut microbiota are affected by a variety of factors during their development, such as species, genetics, and dietary conditions, and even different locations in the gut have an impact on the composition of the microbial community (Kim and Isaacson, 2015; Zhao et al., 2015). In this study, we observed changes in the abundance of some bacteria at the genus level with age. We found that the abundance of *Lactobacillus* was decreased on days 14 and 42 after exposure to the antibiotic, which is in line with the findings of Yu et al. (2018). However, the abundance of *Lactobacillus* was increased on the 164th day of exposure to the antibiotic. We also found that the abundance of *Lactobacillus* was highest in the LR group on day 14 of this study, suggesting that exposure to probiotics early in pigs’ life is beneficial to increase the proportion of *Lactobacillus.* This different response may be due to changes in intestinal physiological conditions and environmental factors as pigs age and, thus, different effects of antibiotics on microbial communities. Besides, we noticed that the proportions of *Ruminococcaceae* were increased with age compared with the control group, and that the abundance of *Ruminococcaceae* in the LR group was decreased, indicating that both of them can affect the abundance of *Ruminococcaceae*. Our results are consistent with previous reports that *L. reuteri* had a negative regulatory effect on *Ruminococcaceae* (Liu et al., 2019a; Zhang et al., 2019). In addition, we observed a significant decrease in the ratio of *Lactobacillus* to *E. coli* in the AO group on days 14 and 42 compared to the control group. The ratio of the LR group was significantly higher compared to the AO group. These results and our results of PCA and PCoA indicate that the microbial community of pig colon in different periods changed dynamically with time, and that these changes may contribute to intestinal development and maturation. Interestingly, we also found that AO and LR had a little effect on cecum predominant bacteria phyla, and that the major bacterial phyla in the AO and LR groups were *Firmicutes*, *Proteobacteria*, and *Bacteroidetes*. Zhang et al. found that differences in microbial alpha diversity between the oral administration of *L. reuteri* and antibiotics were not significant in the pig colon and cecum but were significant in the jejunum (Zhang et al., 2016). Similarly, as observed in this study, AO and LR had no significant effects on the alpha diversity of bacteria in the cecum. Previous studies have shown that the microbial composition of the large intestine is more stable than that of the small intestine (Liu et al., 2019b). In addition, the drugs in the diet had a greater effect on the change of microbe in the foregut but not in the hindgut, and even if exposed to antibiotics early in life, the ecological environment in the intestine will not change much (Donaldson et al., 2016). In this study, we added antibiotics to the feed according to the prescribed safe dosage. We did not find that antibiotics have an adverse effect on intestinal microbes. Therefore, we believed that this is due to differences in the composition and development of the intestinal microbiota in different intestinal segments and possibly related to the gradual dilution of AO and LR in the gut, which led to weakened effects. Furthermore, it may be because antibiotics and *L. reuteri* are secondary variables relative to the ecological environment of the cecum or because the gut matures with age.

To further investigate the effects of the feed with AO or LR and age on the cecum of pigs, we detected the concentration of short-chain fatty acids (SCFAs) in the cecum. The cecum is the main site of SCFA production (Brestenský et al., 2017). SCFAs have been reported to provide energy for the intestinal epithelium and maintain intestinal homeostasis through anti-inflammatory effects (Correa-Oliveira et al., 2016; Fouhse et al., 2016). Interestingly, we found that dietary AO and LR had no significant effects on SCFA concentration in pigs, but that the age of pigs has significant effects on them. Tang et al. found that piglets exposed to lincomycin had a decreased concentration of SCFAs (Tang et al., 2021). This is inconsistent with our results; on the one hand, it may be due to the significant difference in dosage between us and them and the difference in antibiotic types. On the other hand, the intestinal flora of the cecum was not disturbed after antibiotic administration, producing unfavorable bacteria. Members of *Ruminococcaceae* and *Lachnospiraceae* are bacteria that indirectly produce butyrate (Esquivel-Elizondo et al., 2017; Liao et al., 2021). Our results showed that the relative abundance of *Ruminococcaceae* and *Lachnospiraceae* was increased along with age. Franklin et al. showed that pig intestinal SCFA concentrations and microbial populations are influenced by weaning age (Franklin et al., 2002). Recently, Qi et al. also found that total SCFA concentration was increased as pigs aged (from lactation and early nursery; Qi et al., 2020). Hence, we speculated that the influence of age on SCFAs may be related to it. To fully understand the effects of AO and LR on the cecal intestinal environment, it is necessary to fully consider the changes in dose and verify through a large number of samples in the future to make up for the insufficient sample size of this trial.

## Conclusion

In conclusion, neither LR nor AO damaged the intestinal barrier function of pig cecum, and they had little effect on the cecal microbiota in different periods. The microflora and their metabolite SCFAs were significantly changed along with age. These findings provide important information to understand the homeostasis of the cecum of pigs after antibiotic or probiotic treatment. The potential mechanisms of LR and antibiotics regulating cecal intestinal barrier function differences and their effects on microbial communities need to be explored in the future.

## Data Availability Statement

The datasets presented in this study can be found in online repositories. The names of the repository/repositories and accession number(s) can be found below: https://www.ncbi.nlm.nih.gov/, PRJNA768304.

## Ethics Statement

The experiment was carried out in accordance with the Chinese guidelines for animal welfare and experimental protocol, and approved by the Animal Care and Use Committee of Guangdong Academy of Agricultural Sciences.

## Author Contributions

BY and CL contributed to conceptualization and investigation. QW and YH contributed to methodology. YX, XY, and SH contributed to data curation, formal analysis, and software. HY, QW, and YX contributed to validation and visualization. BY and HY contributed to writing the original draft. LW and ZJ contributed to writing, reviewing, editing the manuscript, funding acquisition, project administration, and resources. All authors have read and agreed to the published version of the manuscript.

## Conflict of Interest

The authors declare that the research was conducted in the absence of any commercial or financial relationships that could be construed as a potential conflict of interest.

## Publisher’s Note

All claims expressed in this article are solely those of the authors and do not necessarily represent those of their affiliated organizations, or those of the publisher, the editors and the reviewers. Any product that may be evaluated in this article, or claim that may be made by its manufacturer, is not guaranteed or endorsed by the publisher.
