# Supplementary material for: The Responses of Lactobacillus reuteri LR1 or Antibiotic on Intestinal Barrier Function and Microbiota in the Cecum of Pigs
Source: Front Microbiol. 2022 Jun 2;13:877297. doi: 10.3389/fmicb.2022.877297 (PMC9201390; doi:10.3389/fmicb.2022.877297)
Supplement: Supplementary file 1 [file Table_1.DOCX]

Table.1 The formulations and chemical composition of the basal diet (as-fed basis) at different stages

| Ingredient | 7-11  kg | 11-25  kg | 25-50  kg | 50-75  kg | 75-100  kg | 100-13  kg |
| --- | --- | --- | --- | --- | --- | --- |
| Corn, 8% | 26.2 | 38 | 72.8 | 75.2 | 79 | 80.4 |
| Extrude corn, 8% | 25 | 22 |  |  |  |  |
| Fermented soybean meal, 47% | 14.7 | 14.4 | 16 | 14 | 9.2 | 6 |
| Expanded soybean | 10 | 10 |  |  |  |  |
| [Wheat](D:/%E5%AE%9E%E9%AA%8C%E5%AE%A4/%E6%96%B0%E5%BB%BA%E6%96%87%E4%BB%B6%E5%A4%B9/wangyiyoudaocidian/8.9.9.0/resultui/html/index.html#/javascript:;) [bran](D:/%E5%AE%9E%E9%AA%8C%E5%AE%A4/%E6%96%B0%E5%BB%BA%E6%96%87%E4%BB%B6%E5%A4%B9/wangyiyoudaocidian/8.9.9.0/resultui/html/index.html#/javascript:;) |  |  | 4 | 5 | 6 | 8 |
| Fish meal, 63% | 4 | 4 | 2 |  |  |  |
| Whey powder, 3% | 11 | 6 |  |  |  |  |
| [Plasma](D:/%E5%AE%9E%E9%AA%8C%E5%AE%A4/%E6%96%B0%E5%BB%BA%E6%96%87%E4%BB%B6%E5%A4%B9/wangyiyoudaocidian/8.9.9.0/resultui/html/index.html#/javascript:;) [protein](D:/%E5%AE%9E%E9%AA%8C%E5%AE%A4/%E6%96%B0%E5%BB%BA%E6%96%87%E4%BB%B6%E5%A4%B9/wangyiyoudaocidian/8.9.9.0/resultui/html/index.html#/javascript:;) [powder](D:/%E5%AE%9E%E9%AA%8C%E5%AE%A4/%E6%96%B0%E5%BB%BA%E6%96%87%E4%BB%B6%E5%A4%B9/wangyiyoudaocidian/8.9.9.0/resultui/html/index.html#/javascript:;), 77.8% | 3 |  |  |  |  |  |
| Soybean oil | 0.4 |  | 1.5 | 2 | 2.2 | 2.36 |
| Custer sugar | 2 | 2 |  |  |  |  |
| 50% Choline chloride | 0.1 | 0.1 | 0.08 | 0.08 | 0.06 | 0.06 |
| Salt | 0.25 | 0.25 | 0.3 | 0.3 | 0.35 | 0.35 |
| Monocalcium phosphate | 1 | 0.9 | 0.9 | 1 | 0.8 | 0.55 |
| Limestone | 0.7 | 0.6 | 0.8 | 0.8 | 0.78 | 0.8 |
| L-Lys HCl | 0.41 | 0.51 | 0.45 | 0.43 | 0.43 | 0.37 |
| DL-Met | 0.1 | 0.07 | 0.03 | 0.04 | 0.04 |  |
| L-Thr | 0.14 | 0.17 | 0.14 | 0.15 | 0.14 | 0.11 |
| Premix^1^ | 1 | 1 | 1 | 1 | 1 | 1 |
| Total | 100 | 100 | 100 | 100 | 100 | 100 |
| Energy and nutrient composition |  |  |  |  |  |  |
| DE, kcal/kg | 3549 | 3492 | 3425 | 3424 | 3423 | 3413 |
| CP (analyzed), % | 20.6 | 18.8 | 15.9 | 14.0 | 12.1 | 10.9 |
| Ca (analyzed), % | 0.78 | 0.70 | 0.65 | 0.59 | 0.52 | 0.46 |
| P (analyzed), % | 0.66 | 0.61 | 0.56 | 0.52 | 0.47 | 0.43 |
| STTD of P, % | 0.47 | 0.40 | 0.35 | 0.32 | 0.28 | 0.23 |
| Lys, % | 1.53 | 1.40 | 1.12 | 0.97 | 0.84 | 0.71 |
| Met, % | 0.44 | 0.40 | 0.31 | 0.28 | 0.26 | 0.20 |
| Thr, % | 0.96 | 0.86 | 0.71 | 0.65 | 0.57 | 0.49 |
| Trp, % | 0.25 | 0.21 | 0.17 | 0.15 | 0.12 | 0.11 |

^1^Provided vitamin and mineral premix per kg of diet: vitamin A = 2400 IU; vitamin D3 = 2800 IU; vitamin E = 200 IU; vitamin K3 = 5 mg; vitamin B12 = 40 μg; vitamin B1 = 3 mg; vitamin B2 = 10 mg; niacin = 40 mg; pantothenic acid = 15 mg; folic acid = 1 mg; vitamin B6 = 8 mg; biotin = 0.08 mg; Fe (FeSO4•H2O) = 120 mg; Cu (CuSO4•5H2O) = 16 mg; Mn (MnSO4•H2O) = 70 mg; Zn (ZnSO4•H2O) = 120 mg; I (CaI2O6) = 0.7 mg; Se (Na2SeO3) = 0.48 mg.

Table 2. Primers for the real-time PCR analysis

| **Gene^1^** | **Accession number** | **Sequence (5’-3’)** | **Size(bp)** |
| --- | --- | --- | --- |
| *ZO-1* | [XM_013993251](https://www.ncbi.nlm.nih.gov/nuccore/XM_013993251) | Forward:AGCCCGAGGCGTGTTT | 147 |
|  |  | Reverse: AGCCCGAGGCGTGTTT |  |
| *Occludin* | [XM_005672525](https://www.ncbi.nlm.nih.gov/nuccore/XM_005672525) | Forward: GCACCCAGCAACGACAT | 144 |
|  |  | Reverse:CATAGACAGAATCCGAATCAC |  |
| *Mucin-2* | [XM_007465997.1](https://www.ncbi.nlm.nih.gov/nuccore/XM_007465997.1) | Forward: CTGCTCCGGGTCCTGTGGGA | 100 |
|  |  | Reverse:  CCCGCTGGCTGGTGCGATAC |  |
| *Mucin-1* | XM_021089730.1 | Forward: ACACCCATGGGCGCTATGT | 68 |
|  |  | Reverse: GCCTGCAGAAACCTGCTCAT |  |
| *pBD2* | [AY506573.1](https://www.ncbi.nlm.nih.gov/nuccore/AY506573.1) | Forward: CCAGAGGTCCGACCACTACA | 88 |
|  |  | Reverse: GGTCCCTTCAATCCTGTTGAA |  |
| *PG1-5* | [XM_005669497.2](https://www.ncbi.nlm.nih.gov/nuccore/XM_005669497.2) | Forward: GTAGGTTCTGCGTCTGTGTCG | 166 |
|  |  | Reverse: CAAATCCTTCACCGTCTACCAC |  |
| *GAPDH* | [NM_001206359](https://www.ncbi.nlm.nih.gov/nuccore/NM_001206359) | Forward:ACTCACTCTTCCACTTTTGATGCT | 100 |
|  |  | Reverse: ACTCACTCTTCCACTTTTGATGCT |  |

^1^*ZO-1* = *zonula occludens-1*; *pBD2* = *porcine β defensin 2*; *PG1-5* = *protegrin 1-5*;*GAPDH* = *glyceraldehyde-3-phosphate dehydrogenase*.

Table 3. The sample sequence information

| **Sample** | **PE_reads** | **Nochimera** | **AvgLen(bp)** | **Effective(%)** |
| --- | --- | --- | --- | --- |
| S1_AO1 | 79562 | 64248 | 445.65 | 52.52 |
| S1_AO2 | 82620 | 65895 | 443.90 | 52.66 |
| S1_AO3 | 91073 | 76311 | 447.12 | 52.57 |
| S1_AO5 | 85480 | 66246 | 446.33 | 52.9 |
| S1_AO6 | 82825 | 69206 | 446.02 | 53.01 |
| S1_CON2 | 97846 | 83254 | 446.71 | 52.8 |
| S1_CON3 | 84512 | 67939 | 447.73 | 52.41 |
| S1_CON4 | 98220 | 77372 | 448.08 | 52.89 |
| S1_CON6 | 94966 | 81495 | 445.64 | 54.3 |
| S1_CON7 | 93839 | 76934 | 446.33 | 52.96 |
| S1_LR2 | 74690 | 61230 | 447.82 | 52.66 |
| S1_LR3 | 89714 | 77642 | 450.84 | 52.28 |
| S1_LR4 | 82344 | 66901 | 448.93 | 52.08 |
| S1_LR7 | 81609 | 65401 | 450.73 | 52.54 |
| S1_LR8 | 80805 | 67236 | 446.01 | 53.02 |
| S2_AO2 | 86385 | 69495 | 445.42 | 53.04 |
| S2_AO4 | 94663 | 76165 | 448.00 | 53.2 |
| S2_AO6 | 82846 | 68548 | 446.47 | 52.78 |
| S2_AO7 | 95085 | 77202 | 449.72 | 53.06 |
| S2_AO8 | 82068 | 65821 | 447.42 | 53.15 |
| S2_CON1 | 81117 | 66731 | 448.40 | 53.13 |
| S2_CON3 | 95047 | 80397 | 447.57 | 52.97 |
| S2_CON4 | 93736 | 75429 | 450.71 | 53.21 |
| S2_CON5 | 80114 | 63763 | 445.63 | 52.99 |
| S2_CON6 | 61110 | 49418 | 445.95 | 53.47 |
| S2_LR1 | 102596 | 80177 | 445.79 | 53.07 |
| S2_LR3 | 81755 | 65701 | 445.51 | 52.9 |
| S2_LR4 | 83950 | 64777 | 449.81 | 52.53 |
| S2_LR6 | 78692 | 60190 | 447.48 | 54.41 |
| S2_LR8 | 83080 | 65040 | 451.21 | 52.64 |
| S3_CON1 | 86303 | 74513 | 445.63 | 52.86 |
| S3_CON3 | 83352 | 71380 | 443.53 | 53.34 |
| S3_CON5 | 83414 | 72085 | 448.14 | 52.85 |
| S3_CON7 | 76894 | 67625 | 446.44 | 53.28 |
| S3_CON8 | 82385 | 73371 | 445.63 | 53.06 |
| S3_LR2 | 92310 | 79782 | 444.67 | 53.22 |
| S3_LR5 | 92257 | 81994 | 447.00 | 53.49 |
| S3_LR6 | 79152 | 66918 | 445.04 | 52.99 |
| S3_LR7 | 92134 | 81668 | 444.20 | 53.07 |
| S3_LR8 | 66565 | 55381 | 448.74 | 52.93 |


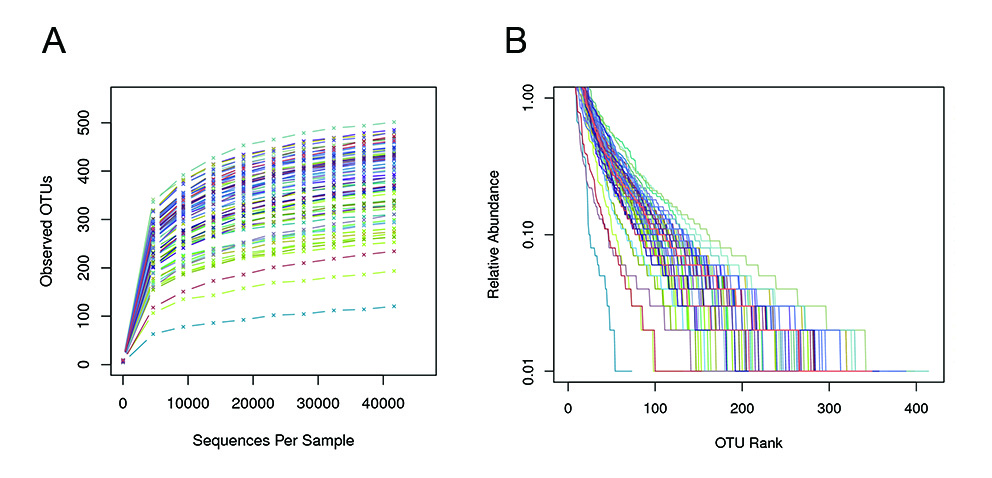


FIGURE 1. Feasibility analysis of cecum samples. the rank-abundance curve (A) and rarefaction curve (B).


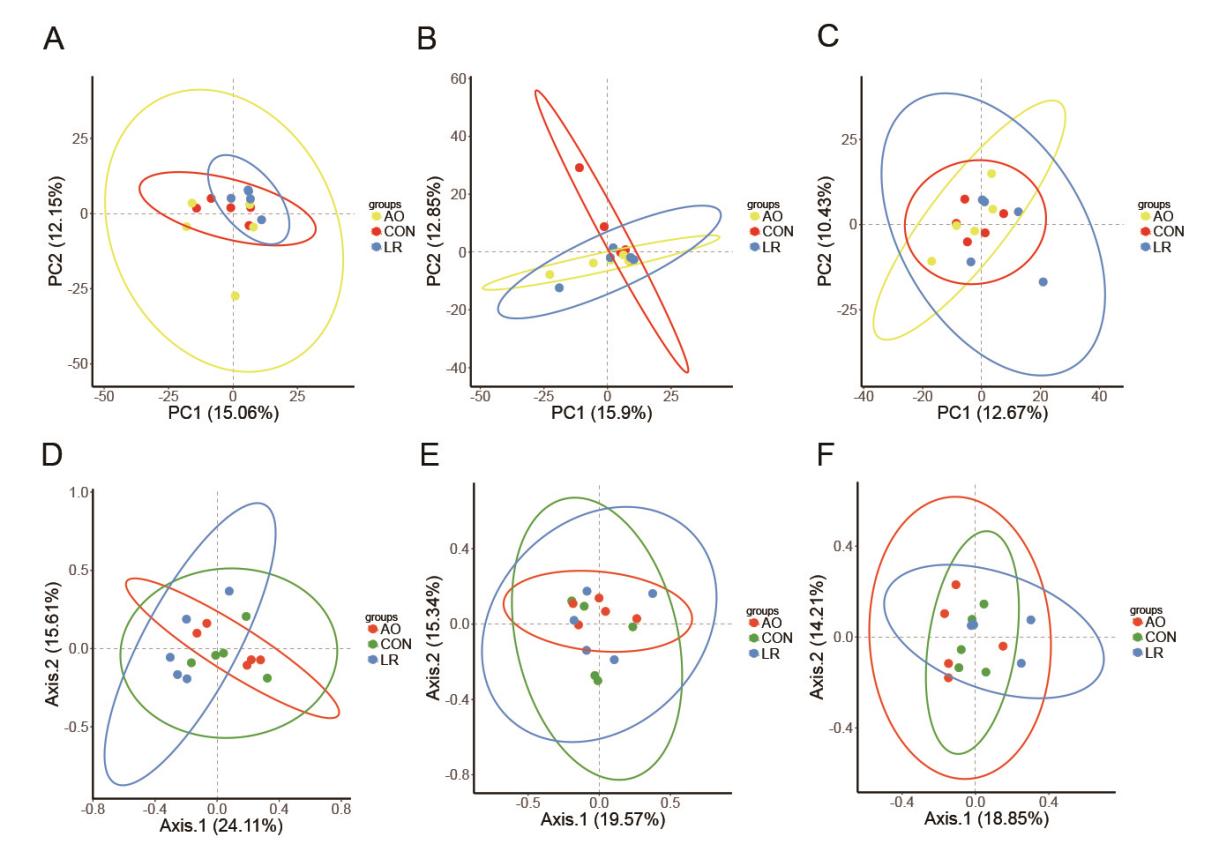


FIGURE 2. Principal component analysis (PCA) and principal co-ordinates analysis (PCoA) of S1 stage cecal microbiota in three groups was performed (A&D). PCA and PCoA of S2 stage in three groups (B&E). PCA and PCoA of S3 stage in three groups (C&F). Each dot represents a sample, and the same color indicates the same group.


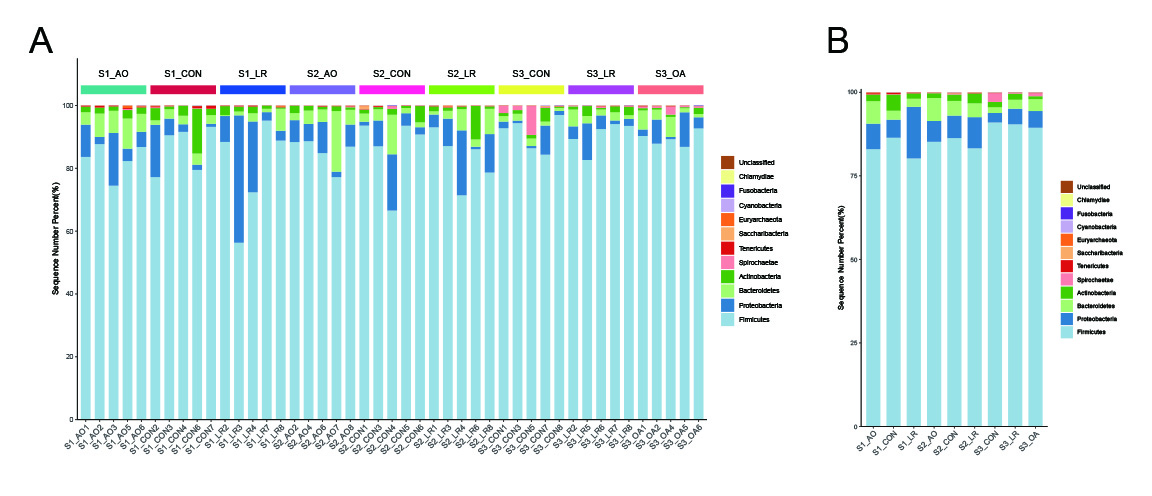


FIGURE 3. Relative abundance of intestinal microorganisms. Relative abundance of the most dominat cecal microbiota in each sample at the phylum levels (A). Relative abundance of cecal microbiota on the basis of the average number of each subfamily at the phylum (B).
